# Two Signals from One Event: Exploiting Intrinsic Dual-Functional Selenium Nanomaterials for Signal-On Chiroptical and Colorimetric Sensing of Free Thiols

**DOI:** 10.3390/bios16090493

**Published:** 2026-09-03

**Authors:** Jiayang Gao, Xinling Liu

**Affiliations:** 1 College of Food Science and Technology, Shanghai Ocean University, Shanghai 201306, China; 2334202@st.shou.edu.cn; 2 The Education Ministry Key Lab of Resource Chemistry, Shanghai Frontiers Science Center of Biomimetic Catalysis, College of Chemistry and Materials Science, Shanghai Normal University, Shanghai 200234, China

**Keywords:** free thiols, colorimetric, circular dichroism, selenium nanomaterials, chiral

## Abstract

Rapid identification of free thiols is essential in pharmaceutical quality control and food safety. Herein, we demonstrate the sensing concept of “two signals from one event” by exploiting the intrinsic dual functionality of selenium (Se) nanomaterials to construct a signal-on, dual-mode sensing platform for free thiols. The single event is thiol-triggered seeded growth: free thiols interact with L-cysteine-modified Se seeds, driving seed fusion and crystallization into chiral trigonal Se structures. This same event yields two readouts simultaneously: a colorimetric response arising from particle size enlargement (UV-Vis red-shift) and a turn-on circular dichroism (CD) signal from long-range chiral ordering. The method achieves a detection threshold of 10 μmol/L for various free thiols and can be completed within 60 min from seed preparation to signal readout without complex instrumentation. It serves as a general indicator of total free thiols rather than distinguishing individual thiol species. Above the threshold, concentration-dependent color deepening and spectral redshifts provide semi-quantitative estimation of the concentration range. Its practicality was validated by detecting D-penicillamine in commercial tablets. This strategy offers an approach for on-site free thiol screening and highlights the potential of chiral inorganic nanomaterials in multi-mode sensing via chiral transfer and amplification.

## 1. Introduction

Free thiols (-SH) are among the most chemically reactive functional groups, and low-molecular-weight thiols such as L-cysteine (L-Cys), D-penicillamine (D-Pen), and L-glutathione (L-GSH) are of particular importance in various industries and biological systems [1,2]. For example, L-Cys is widely used as a flour improver, antioxidant, and nutritional supplement, and D-Pen is a chelating agent for Wilson’s disease and a pharmaceutical raw material. Given their extensive applications, rapid and reliable identification of free thiols is critical in many areas, including pharmaceutical quality control and food safety.

Conventional analytical techniques for thiols (e.g., high-performance liquid chromatography, fluorometric assays) offer high sensitivity but require skilled operation, time-consuming sample pretreatment, and multistep synthesis of fluorescent probes and are not amenable to on-site quick screening [1,3,4,5]. Nanomaterial-based colorimetric assays provide a simpler alternative, yet most rely on a single readout, limiting reliability and confirmatory capacity [2,6,7,8,9]. These limitations underscore the need for multi-mode sensing strategies that can deliver complementary information within a single assay.

Notably, L-Cys, D-Pen, and L-GSH are inherently chiral, making circular dichroism (CD) spectroscopy a natural detection approach. However, the CD signals of small molecules are intrinsically weak and confined to the UV region, requiring high concentrations for observable signal output. A promising solution to overcoming this limitation lies in chiral interactions at inorganic–organic interfaces. When chiral molecules bind to the surface of inorganic nanomaterials through specific interactions, their molecular chirality can be transferred to the inorganic hosts, giving rise to induced CD signals that are often red-shifted to the visible region and significantly enhanced in intensity compared with those of the original chiral molecules [10,11,12,13,14]. Such chirality transfer and amplification at inorganic–organic interfaces provide a foundation for harnessing CD spectroscopy as a practical readout for detecting thiols.

Selenium (Se) nanomaterials are particularly attractive in this regard. Unlike conventional nanoparticle probes that typically rely on external labels or exploit only a single optical property, Se nanomaterials inherently combine two distinct optical functionalities within one material. First, trigonal selenium (t-Se) exhibits a size- and morphology-dependent absorption band in the visible region, making it inherently suitable for colorimetric readout [15,16]. Second, its crystal structure—belonging to the chiral space groups P3_1_21 or P3_2_21—consists of helical chains of Se atoms that generate characteristic CD signals [17,18,19,20]. Therefore, it is anticipated that this intrinsic dual functionality may allow the same nanomaterial system to serve as both a colorimetric probe and a chiroptical sensor without additional modification. However, the chiroptical activity of t-Se is highly dependent on crystallinity and long-range order; small or poorly crystalline seeds often exhibit negligible CD signals despite possessing a chiral lattice. This suggests that a chiral amplification strategy—enhancing weak chiroptical responses through seeded growth—could be exploited to construct signal-on chiroptical sensors.

**Scheme 1:**
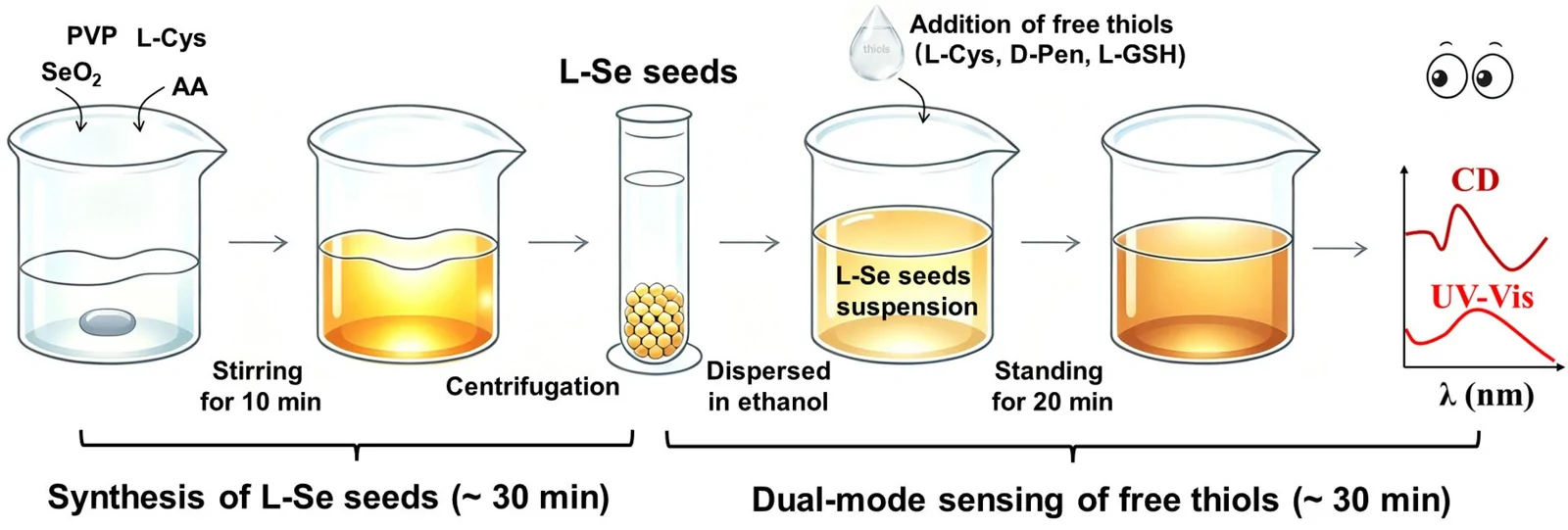
Schematic illustration of the synthesis of L-Se seeds and their application in chiroptical and colorimetric dual-mode sensing of free thiols.

Herein, we report a signal-on, dual-mode sensing platform for the threshold identification of free thiols, based on thiol-triggered chiral amplification at selenium seeds (Scheme 1). This design embodies the concept of “two signals from one event”: the same event—thiol-triggered seeded growth via Se-S bond formation—simultaneously yields visual color changes (due to particle size enlargement) for rapid, instrument-free screening and turn-on CD signals (due to chiral crystallization) for sensitive chiroptical confirmation. The entire procedure—from seed preparation to detection—can be completed within 60 min using low-cost reagents and achieves a detection threshold down to 10 μmol/L, making it suitable for rapid, on-site screening applications.

## 2. Materials and Methods

Materials: Selenium dioxide (SeO_2_), ascorbic acid (AA), polyvinylpyrrolidone (PVP, Mw: 30,000–40,000), L-cysteine (L-Cys), D-penicillamine (D-Pen), L-glutathione (L-GSH), L-valine (L-Val), L-phenylalanine (L-Phe), L-methionine (L-Met), L-histidine (L-His), D-glucose (D-Glu), D-sucrose (D-Suc), zinc acetate dihydrate, copper (II) sulfate pentahydrate, and ethanol were purchased from Shanghai Titan Scientific Co., Ltd (Shanghai, China). All water used was ultrapure.

Synthesis of L-Se seeds: The synthesis of L-Se seeds was carried out according to a previously reported procedure [20], with minor modifications. As shown in Scheme 1, an aqueous solution of SeO_2_ (26.67 mM, 5 mL) was mixed with PVP (3.33 mg/mL, 5 mL) under stirring at room temperature. L-Cys solution (6 mM, 5 mL) was then added, followed by the addition of the AA solution (53.33 mM, 5 mL). The mixture was stirred for 10 min, during which the color changed to yellow. The resulting suspension was centrifuged at 8000 rpm for 5 min, and the supernatant was discarded. The collected precipitate (denoted by L-Se seeds) was washed once by resuspension in ultrapure water with the aid of ultrasonication, followed by a second centrifugation to remove residual reagents.

CD and colorimetric dual-mode identification of free thiols: As displayed in Scheme 1, the above washed L-Se seeds were subsequently dispersed in 50 mL of ethanol to serve as the seed stock solution. Using L-Cys as the typical thiol, the thiol-triggered crystallization was conducted as follows: Briefly, 2 mL aliquots of the above seed suspension were mixed with L-Cys solutions (0.5 mL) of different concentrations (0–40 μmol/L). The mixtures were allowed to stand at room temperature for 20 min, and the resulting suspensions were used directly for CD and UV-Vis absorption spectroscopic measurements without further purification, using a 4:1 (*v*/*v*) ethanol/water mixture for baseline correction.

Characterizations: UV-Vis absorption and CD spectra were measured and collected on a J-1500 spectrophotometer (JASCO, Tokyo, Japan). Transmission electron microscopy (TEM) and energy-dispersive X-ray (EDS) spectroscopy were performed on a JEM-F200 microscope (JEOL, Tokyo, Japan) operated at 200 kV. X-ray diffraction (XRD) patterns were collected on a SmartLab diffractometer (Rigaku, Tokyo, Japan). Raman spectra were acquired on a Super LabRam II Raman microscope (HORIBA, Palaiseau, France) with a 633 nm excitation laser.

## 3. Results

### 3.1. Preparation and Characterization of L-Se Seeds

As outlined in Scheme 1, the synthesis of L-Se seeds was achieved by reducing SeO_2_ with AA, while PVP was included to ensure good dispersion of the seeds. Notably, L-Cys was introduced as a surface modifier, leveraging its ability to form stable Se-S bonds with the selenium surface. This surface functionalization is critical for the subsequent chiroptical and colorimetric identification of free thiols, as will be elaborated in detail later.

**Figure 1:**
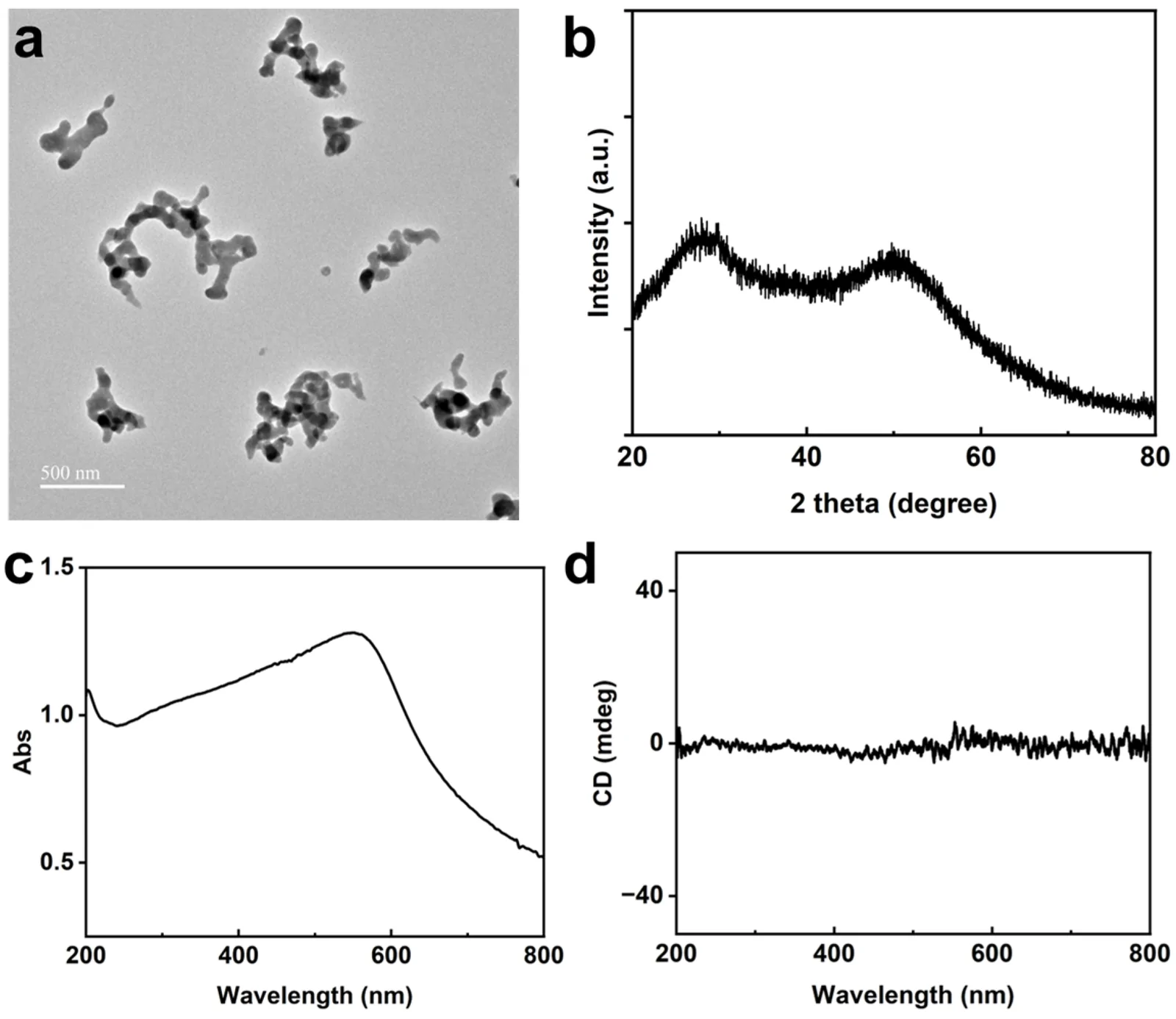
Characterization of L-Se seeds: (**a**) TEM image, (**b**) XRD pattern, (**c**) UV-Vis absorption, and (**d**) CD spectra.

The as-obtained L-Se seed suspension exhibited a yellow color, which is typical for Se nanomaterials [15,16]. In the TEM image (Figure 1a), L-Se seeds appeared as nanoparticles interconnected by ribbon-like structures. Moreover, elements Se, S, and O were detected by EDS spectroscopy (Figure S1), suggesting the presence of L-Cys molecules remaining on the surface of L-Se seeds. The XRD pattern of L-Se seeds is presented in Figure 1b, revealing an amorphous nature as evidenced by the absence of sharp diffraction peaks. The UV-Vis absorption spectrum of L-Se seeds (Figure 1c) displays a broad absorption band in the range of 200–800 nm, with a peak centered at approximately 550 nm. The CD spectrum of L-Se seeds (Figure 1d), however, shows no discernible signals between 200 and 800 nm, indicating that L-Se seeds are CD inactive.

### 3.2. Colorimetric and Chiroptical Dual-Mode Responses to Free Thiols

**Figure 2:**
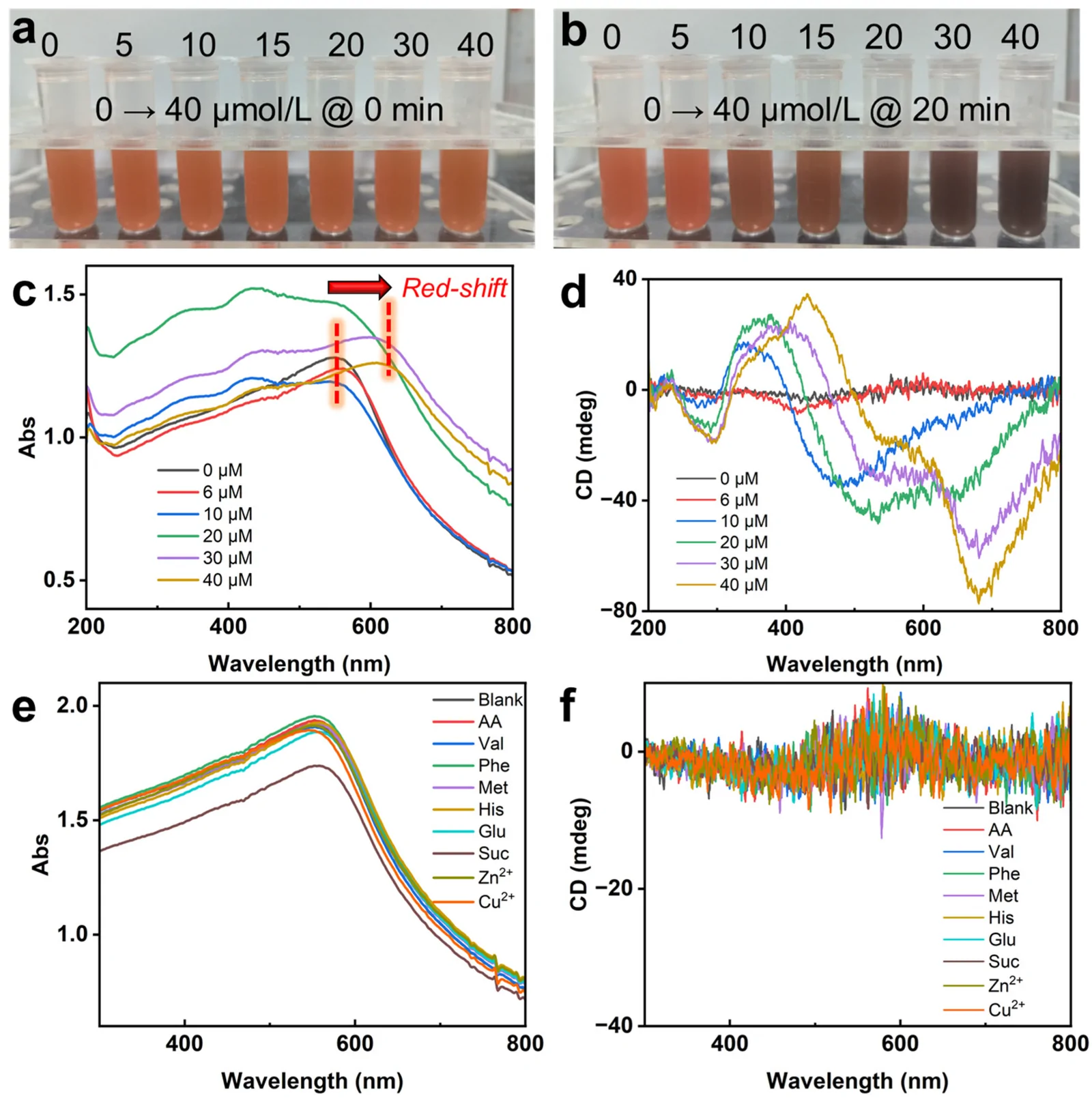
Photographs of L-Se seeds mixed with L-Cys solutions of various concentrations at (**a**) 0 min and (**b**) 20 min, and their corresponding (**c**) UV-Vis absorption and (**d**) CD spectra after 20 min; (**e**) UV-Vis absorption and (**f**) CD spectra of L-Se seeds incubated with various interferents for 20 min.

L-Cys, a representative thiol-containing compound, was employed to investigate the optical response of L-Se seeds toward thiols. A series of L-Cys solutions with varying concentrations were mixed with the L-Se seed suspension and then allowed to stand at room temperature for 20 min. As the concentration of L-Cys (denoted by c_L-Cys_) increased from 0 to 40 μmol/L, the colors of the above mixtures gradually changed from pale yellow to orange-yellow and finally to reddish-brown, indicating a c_L-Cys_-dependent response (Figure 2a–b). Especially, distinct color changes were observable to the naked eye (Figure 2b) when c_L-Cys_ reached 10 μmol/L. At higher concentrations over 10 μmol/L, the coloration progressively deepened to brown. On the basis of these visual observations, L-Cys could be identified at a concentration as low as 10 mmol/L by naked-eye detection.

Furthermore, UV-Vis absorption (Figure 2c) and CD spectroscopy (Figure 2d) were employed in parallel to monitor the L-Cys-induced changes of L-Se seeds. Over the concentration range of c_L-Cys_ from 0 to 10 μmol/L, the UV-Vis absorption spectra remained largely similar, all exhibiting a main peak around 553 nm. However, a redshift was observed when c_L-Cys_ reached 20 μmol/L, and the peak further shifted to approximately 610 nm when c_L-Cys_ was 30 and 40 μmol/L. Concurrently, the solution color turned brown at 20 μmol/L and became increasingly deeper brown at higher concentrations, consistent with the redshift of the absorption bands.

As shown in Figure 2d, the as-prepared L-Se seeds exhibited no CD activity in the absence of L-Cys. Remarkably, even at a low c_L-Cys_ of 5 μmol/L, a broad negative CD signal centered at 418 nm emerged. With progressively increasing c_L-Cys_, the CD signals intensified and developed into multiple distinct bands. In general, the CD profiles featured a negative peak (denoted as peak A) in the short-wavelength region (below 350 nm), followed by a positive peak (peak B, 350–500 nm), and finally a negative peak (peak C) in the long-wavelength visible region. These CD curves are similar to those of chiral Se nanomaterials in previous reports [18,19]. Notably, both peaks B and C exhibited a progressive red shift with increasing c_L-Cys_.

It is worth noting that the sample with c_L-Cys_ of 5 μmol/L displayed a color nearly identical to that of the L-Se seed sample (Figure 2b), and no appreciable redshift was discernible in the UV-Vis spectra between these two samples. Thus, they could not be readily distinguished by the naked eye or by absorption spectroscopy alone. However, they were clearly differentiated by CD spectroscopy: the sample with the addition of 5 μmol/L L-Cys became CD-active, whereas the L-Se seeds remained CD-silent. Therefore, CD spectroscopy offers a complementary analytical tool for the detection of L-Cys at a lower concentration beyond the colorimetric method.

In addition to L-Cys, two other thiol-containing molecules, namely D-Pen and L-GSH, were also examined under identical conditions. As shown in Figure S2, the color changes in L-Se seeds upon the addition of D-Pen solutions at varying concentrations (0–40 μmol/L) were similar to those observed in the case of L-Cys. Specifically, D-Pen could be visually detected at a concentration of 10 μmol/L, with the brownish hue intensifying progressively at higher concentrations, accompanied by a redshift in the UV-Vis absorption spectra. Meanwhile, CD signals became discernible at a D-Pen concentration of 5 μmol/L, and these signals grew stronger and evolved into multiple distinct bands as the concentration increased. In parallel, as depicted in Figure S3, comparable trends in both UV-Vis absorption and CD spectra were also observed for L-GSH over the same concentration range (0–40 μmol/L). Therefore, the current method is unable to discriminate among different thiol species. Instead, this assay is suitable for measuring total free thiols rather than individual thiol species. Taken together, these results demonstrate that L-Se seeds could serve as a versatile probe for the detection of free thiols, providing complementary readouts through both colorimetric and CD spectroscopic methods.

### 3.3. Selectivity of the Dual-Mode Assay for Free Thiol Identification

To further evaluate the selectivity of this method toward free thiols, nine kinds of potential interferents were examined, including ascorbic acid (AA), amino acids (e.g., Phe, Met, Val, His), sugars (e.g., Glu, Suc), and metal ions such as Cu^2+^ and Zn^2+^. The concentration of each interferent was fixed at 100 μmol/L, which is ten times higher than the threshold concentration (10 μmol/L) at which color changes become detectable upon thiol addition. As shown in Figure 2e, the UV-Vis absorption spectra of L-Se seeds in the presence of these interferents overlapped well with that of pristine L-Se seeds, indicating negligible spectral perturbations. Meanwhile, no CD signals were induced by any of these interferents (Figure 2f). It should be noted that the amino acid of Met also contains a sulfur atom, but it exists as a methylthio group (-SCH_3_), whereas the sulfur atom is present as a thiol group in L-Cys, D-Pen, and L-GSH. Therefore, these control experiments confirmed that the present assay exhibited high selectivity toward free thiols.

However, if all the above interferents (including Zn^2+^ and Cu^2+^) were mixed with L-Cys, neither color change nor CD signal was observed (Figure S4a). When Zn^2+^ and Cu^2+^ were excluded from the mixture while keeping all other interferents, both color change and CD signal were observed (Figure S4b). It should be noted that this is not an interference in the conventional sense, but rather a reflection of the selectivity of this method toward the free thiol form. We interpret this result as follows: Zn^2+^ and Cu^2+^ are known to form stable complexes with thiol-containing molecules. In the presence of these metal ions at concentrations far exceeding that of L-Cys, the free thiol groups are effectively sequestered through chelation. Consequently, our method specifically detected free thiols (those with accessible -SH groups capable of interaction with the Se seed surface), while thiols that are already complexed with metal ions did not generate optical responses.

### 3.4. Validation with Commercial Pharmaceutical Samples

As demonstrated above, this dual-mode assay is effective for free thiol detection at concentrations as low as 10 μmol/L, which may serve as a threshold-based preliminary screening tool for rapid “yes/no” decisions. Many practical scenarios—such as checking the thiol levels in pharmaceutical raw materials and food processing or verifying batch-to-batch consistency—require only such “yes/no” judgment rather than detailed precise quantification. Furthermore, when the thiol concentration increased from 10 μM to 40 μM, both the colorimetric and CD channels exhibited concentration-dependent trends: (i) the color gradually deepened from pale yellow to reddish-brown, with the UV-Vis absorption peak progressively redshifting, and (ii) the CD signal intensity increased, and its peak position also shifted to longer wavelengths. Although these shifts did not follow a simple linear relationship with concentration, they allowed for an approximate estimation of the concentration range to provide semi-quantitative information within the concentration range above the threshold to some degree.

**Figure 3:**
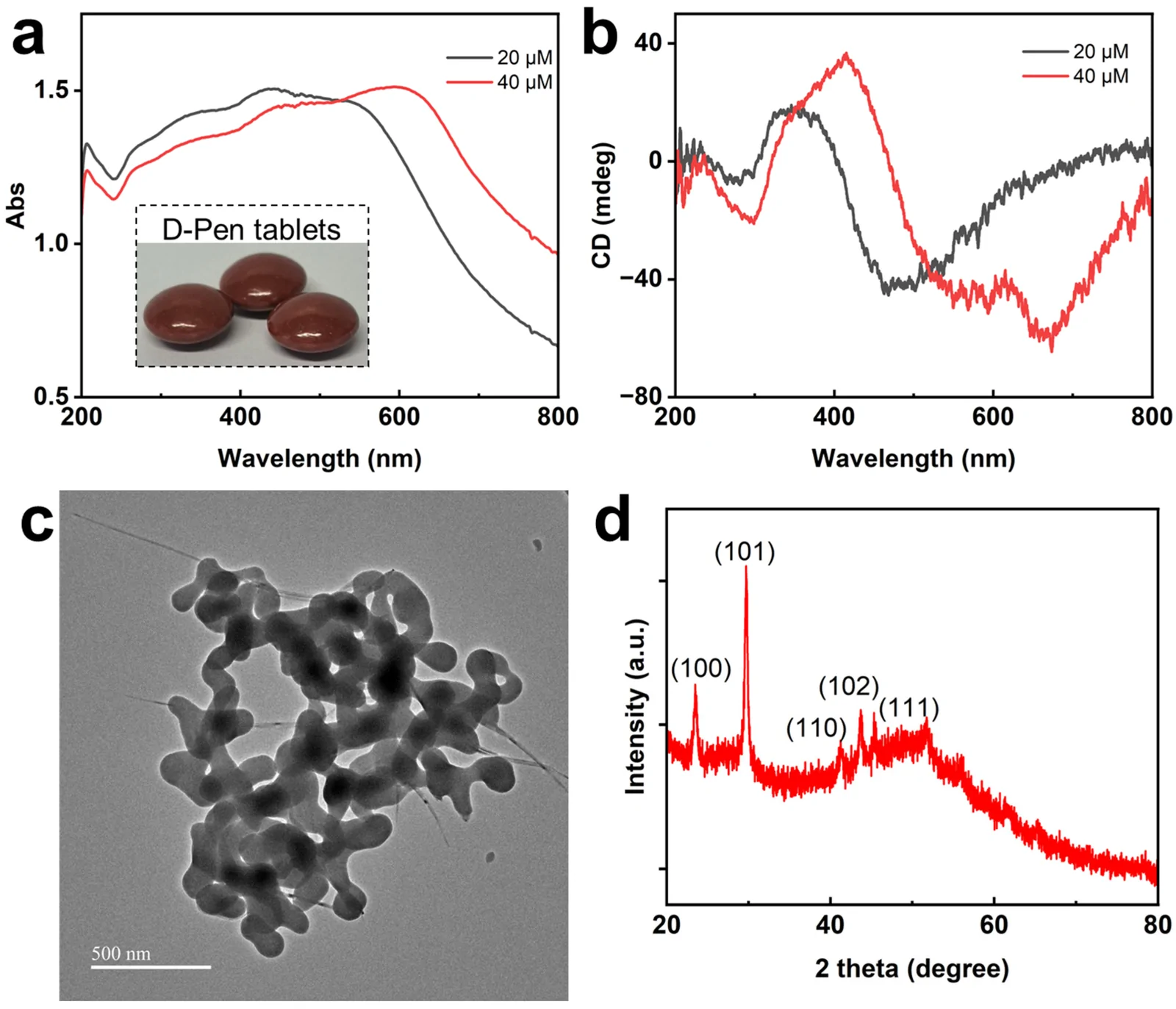
(**a**) UV-Vis absorption (inset, a photograph of commercial D-Pen tablets) and (**b**) CD spectra of L-Se seeds mixed with D-Pen solutions (prepared from D-Pen tablets) at nominal D-Pen concentrations of 20 μmol/L (black lines) and 40 μmol/L (red lines) after 20 min; (**c**) TEM image and (**d**) XRD pattern of the product obtained by mixing L-Se seeds with a D-Pen solution (20 μmol/L) after 20 min.

To validate the practical utility of this method, commercially available D-Pen tablets (Figure 3a, inset) were tested. According to the manufacturer’s specifications, each tablet contains 125 mg of D-Pen, with a total tablet weight approximately twice that amount, indicating the presence of excipients. A tablet was ground, dissolved in water, and then filtered to obtain a clear stock solution containing D-Pen, which was diluted to two different solutions with nominal D-Pen concentrations of 20 and 40 μmol/L. As shown in Figure 3a, with increasing concentration from 20 to 40 μmol/L, we observed similar progressive color deepening and UV-Vis redshift to those seen with standard D-Pen solutions (Figure S2c). The CD signals (Figure 3b) also exhibited similar concentration-dependent peak shifts and intensity enhancements, consistent with the trends observed for pure D-Pen standards (Figure S2d). These results confirm that the semi-quantitative concentration estimation is not limited to pure standard solutions but is also applicable to real pharmaceutical samples containing excipients.

## 4. Discussion

To elucidate the detection mechanism, several control experiments were conducted to clarify the distinct roles of ethanol and L-Cys. To understand the role of ethanol, L-Se seeds were dispersed in pure water rather than ethanol. However, no color change was observed within 20 min after mixing with L-Cys in the pure water solvent. When Se seeds were prepared without the addition of L-Cys, they exhibited no colorimetric or chiroptical response toward free thiols under otherwise identical conditions. Considering L-Cys and D-Cys are typical chiral molecules, we also probed whether L-Se seeds could be used for enantiomeric discrimination. However, D-Cys produced similar colorimetric and CD responses to L-Cys (Figure S5). Similarly, D-Pen also showed similar optical responses to L-Pen. These results confirmed that our method did not distinguish between enantiomers but responded universally to free thiols. Furthermore, we investigated the origin of chiroptical signals through a control experiment. When D-Cys was used instead of L-Cys to prepare the Se seeds (denoted as D-Se seeds), the CD signals of the final products were mirror-symmetric to those obtained from L-Se seeds under otherwise identical conditions (Figure S6). This result indicated that the chirality of the final products is primarily determined by the chiral inducer (L-Cys or D-Cys) introduced during the Se seed preparation stage, rather than by the chirality of the thiols added during the growth stage. These results collectively indicated that both the high ethanol content and the presence of surface-bound L-Cys on the seeds are indispensable for the rapid response.

In addition, the morphology and crystallization degree of the product obtained by mixing L-Se seeds with D-Pen (20 μmol/L) for 20 min were characterized. On the TEM image (Figure 3c), large interconnected aggregates (up to the micrometer scale) formed via coalescence of irregular primary fragments were observed. Therefore, the addition of free thiols would induce an increase in particle sizes and changes in morphologies of Se nanostructures compared with the initial L-Se seeds (Figure 1a). The XRD pattern of the as-obtained product (Figure 3d) is consistent with trigonal Se (t-Se), and its characteristic diffraction peaks are indexed to the (100), (101), (110), (102), and (111) crystal faces.

Based on these observations, the sensing principle of this colorimetric and chiroptical dual-mode assay can be ascribed to a synergistic interplay between thermodynamic driving forces and kinetic facilitation, which together trigger an accelerated Ostwald ripening process that ultimately produces two distinct yet complementary signals. In this section, this mechanism is discussed through three interconnected aspects: (1) the thermodynamic role of ethanol, (2) the kinetic function of thiol-containing molecules as promoters of atomic migration and seed fusion, and (3) the consequent chiroptical signal amplification arising from the formation of crystalline Se products with intrinsically chiral crystal structures.

Ethanol plays a critical role in establishing the thermodynamic conditions necessary for rapid crystallization. It has been documented that ethanol acts as a poor solvent for elemental selenium [20]. By dispersing the L-Se seeds in ethanol, the solubility of Se^0^ is drastically reduced, thereby creating a state of high supersaturation that provides the essential driving force for nucleation and crystal growth. The indispensability of this effect was confirmed by the control experiment in which water was used as the solvent; under this condition, no color change could be observed, underscoring that the high supersaturation induced by ethanol is a prerequisite for the rapid crystallization event.

The role of thiol-containing molecules (e.g., L-Cys, D-Pen, L-GSH) is predominantly kinetic and is mediated through specific surface interactions. It is well established that thiols can interact with selenium to form stable Se-S bonds. In our system, the characteristic -SH stretching band of L-Cys in the Raman spectra (located between 2500 and 2600 cm^−1^, Figure S7) was absent in both the L-Se seeds and the final products after mixing with L-Cys [21], confirming the formation of Se-S bonds. Previous theoretical studies have demonstrated that L-Cys-modified Se clusters exhibit higher adsorption energy for subsequent L-Cys molecules, indicating a significant synergistic enhancement effect at the pre-adsorbed seed interface [20]. Crucially, the adsorbed thiol layer also promotes the migration and rearrangement of selenium atoms, a phenomenon that has been observed in some metal and semiconductor systems. It facilitates the dissolution of smaller, less stable seeds or high-curvature regions on larger seeds, releasing free selenium atoms into the solution; these atoms subsequently diffuse through the liquid phase and redeposit onto the surfaces of larger, more thermodynamically stable seeds [22,23,24,25]. This entire process is a classic manifestation of Ostwald ripening, which is kinetically accelerated by the chemical activity of the adsorbed thiols [26]. The specificity of this mechanism for thiol-containing molecules is further supported by the observation that non-thiol compounds (Figure 2e,f) failed to induce any color change even at concentrations substantially higher than the L-Cys detection threshold, confirming that specific thiol–surface interactions are essential.

The synergy between the high supersaturation created by ethanol and the thiol-mediated surface activation significantly accelerates the Ostwald ripening process, driving seed fusion and growth. This acceleration leads to three critical outcomes. First, smaller seeds dissolve and fuse into larger ones, resulting in a notable increase in the average particle size. Second, this size increase causes a redshift in the ultraviolet–visible absorption spectra, which is visually perceived as a color change constituting the colorimetric signal [27]. Third, the initial Se seeds may possess an intrinsic, albeit weak, chirality due to the use of chiral L-Cys molecules during their synthesis. However, this chirality is not manifested as a detectable CD signal in the small seeds because of their limited particle size and predominantly amorphous nature (Figure 1b). The trigonal crystal structure of selenium consists of helical chains arranged in the chiral space groups P3_1_21 or P3_2_21, which enables the appearance of CD signals in the visible region. As the Ostwald ripening-driven growth proceeds, the increase in particle size and crystallinity allows this intrinsic chiral structure to become more perfectly established over a longer range, resulting in a significant amplification of the chiroptical activity. Consequently, the CD signal emerges and intensifies at wavelengths exceeding 500 nm. This observation indicates that L-Cys in the seed formation stage provides the primary chiral template on the seed surface, while the thiols present during the growth stage act predominantly as growth promoters and surface mediators.

It should be noted that the colorimetric channel requires a thiol concentration of at least 10 μM to produce a naked-eye detectable color change, while the CD channel already generates discernible signals at 5 μM (Figure 2a–d). This difference may arise from the distinct physical origins of the two signals: the colorimetric response depended on particle size enlargement and aggregation, which needed a higher thiol concentration to reach a critical size, whereas the CD signal originated from the intrinsic chirality of the trigonal Se crystal structure. The establishment of local chiral structures may occur at an earlier stage of crystallization to induce CD signals, even when the overall particle size enlargement is not yet sufficient to produce a visible color change. As a consequence, the colorimetric channel (≥10 μM) serves as a rapid, instrument-free preliminary screening tool, while the CD channel (≥5 μM) provides more sensitive confirmatory detection when the colorimetric response is ambiguous. This dual-mode design ensures that both channels are used in a complementary way, with the colorimetric channel offering speed and simplicity and the CD channel providing higher sensitivity for confirmation.

**Scheme 2:**
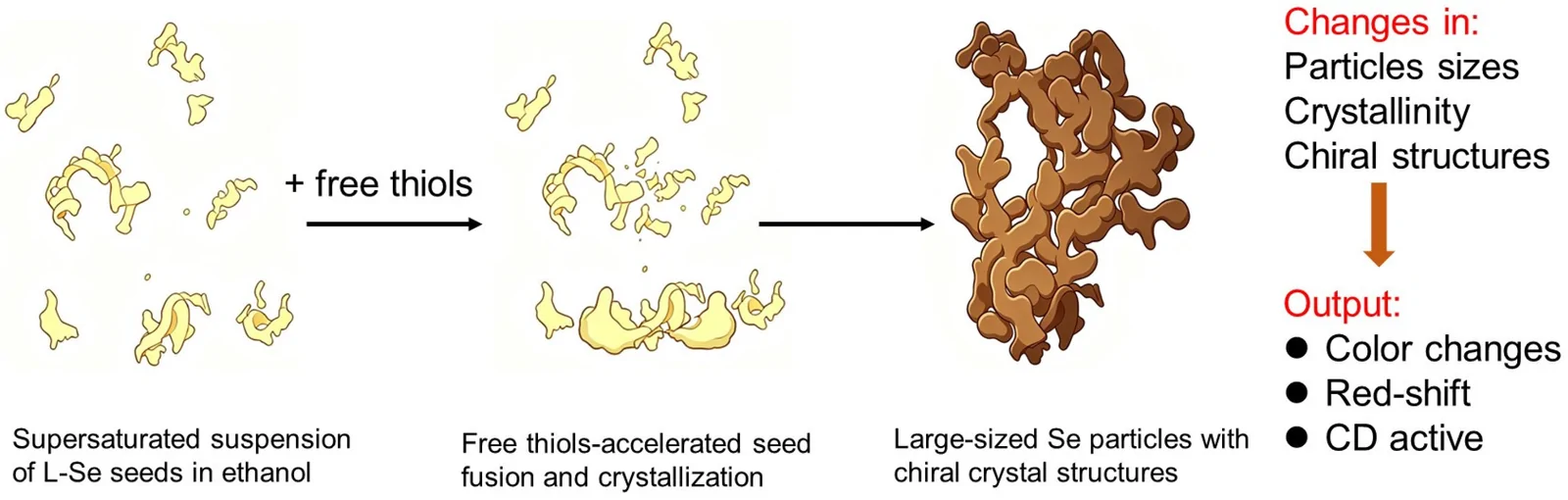
Proposed mechanism for dual-mode identification of free thiols via thiol-triggered fusion and chiral crystallization of L-Se seeds under supersaturated conditions.

As schematically summarized in Scheme 2, the overall detection mechanism can be understood as a cascade: high ethanol content creates a supersaturated environment that thermodynamically favors crystallization; thiol-containing molecules adsorb onto the seed surface via Se-S bonds, reducing the activation energy and promoting Ostwald ripening; this accelerated ripening drives seed fusion and the growth of chiral crystal structures, simultaneously producing a visual colorimetric response through particle size increase and a significantly amplified chiroptical signal through the establishment of long-range chiral order. The dual-mode nature of this assay thus provides both a rapid visual screening capability and a sensitive confirmatory readout within a single experimental platform.

Based on the results and discussion above, the current method is considered a threshold-based screening tool rather than a precise quantitative assay. In many practical applications, a rapid “yes/no” decision on whether the analyte level exceeds a preset threshold is often more valuable than an exact concentration value. The key parameters for threshold detection have been defined: the visual detection threshold is 10 μM, while the CD spectroscopic readout provides instrument-level sensitivity at 5 μM. However, the optical signals were manifested as peak shifts in UV-Vis absorption and CD peak positions, and it is difficult to establish a conventional linear concentration-dependent relationship for quantitative determination. This nonlinearity likely originated from the complexity of the nucleation and seeded-growth process, where the optical responses reflected structural evolution in particle size and chiral structures. Nevertheless, we would like to emphasize that semi-quantitative information may be accessible above the threshold, as indicated by the progressive color deepening and the concomitant redshifts of UV-Vis absorption and CD peaks with increasing thiol concentration (Figure 2b–d). This semi-quantitative capability was further validated in the analysis of commercial D-Pen tablets (Figure 3a–b).

In this context, our method offers several advantages: a visual detection threshold of 10 μM (with CD detection at 5 μM), a total assay time of 60 min, low-cost reagents, and no requirement for specialized instrumentation for the colorimetric readout. The combination of threshold-based “yes/no” judgment with semi-quantitative range estimation further enhances its practical utility for on-site screening applications.

However, several limitations of the current method should be acknowledged. First, it does not distinguish among different thiol species, as all free thiols trigger the same seeded growth event via Se–S bond formation. Second, it is not suitable for precise quantitative analysis. Third, the method specifically detects free thiols rather than total thiol content; thiols that are complexed with metal ions or engaged in disulfide bonds do not generate a response. These characteristics, however, stem from the principle of the assay: it is intended as a rapid screening tool for free thiols in scenarios where the thiol type is already specified or only total free thiol content is of interest. For samples requiring speciation or total thiol determination, complementary techniques or appropriate sample pretreatment would be necessary.

To overcome the above limitations, several directions for future improvement are envisioned. To enable differentiation among thiol species, surface modification of the Se seeds with functional groups that confer selective affinity toward specific thiols could be explored. To establish quantitative calibration, systematic investigation of key parameters governing the nucleation and growth process may enable the construction of a more predictable relationship between optical response and thiol concentration. In addition, sample pretreatment protocols could be developed to extend the method to total thiol detection.

## 5. Conclusions

In summary, we have demonstrated the concept of “two signals from one event” by exploiting the intrinsic dual functionality of Se nanomaterials—size-dependent absorption and chiral crystal structures—to construct a signal-on, dual-mode sensing platform for free thiols. The same thiol-triggered seeded growth event simultaneously yields a colorimetric response (from particle size evolution) and a turn-on CD signal (from long-range chiral ordering), enabling both visual screening and chiroptical confirmation. The assay achieves a detection threshold of 10 μmol/L to offer a clear “yes/no” answer within 60 min using low-cost reagents and provides semi-quantitative concentration estimation above the threshold through concentration-dependent color deepening and spectral red-shifts. It responds universally to free thiols, serving as a general indicator of total free thiols rather than distinguishing individual thiol species. Validation with commercial D-Pen tablets confirmed its practicality. Instead of integrating multiple probes for different signals, this work demonstrates that a single material without external labeling can provide two complementary readouts by virtue of its intrinsic properties, offering a paradigm for multi-mode sensing that may inspire broader applications of chiral inorganic nanomaterials.

## Data Availability

The original contributions presented in this study are included in the article/Supplementary Materials. Further inquiries can be directed to the corresponding author.

## Supplementary Materials

The following supporting information can be downloaded at https://www.mdpi.com/article/10.3390/bios16090493/s1, Figure S1: EDS spectrum of L-Se seeds (the atomic ratio of Se, S, O is 97.49%, 0.27%, and 0.38%, respectively); Figure S2: Photographs of L-Se seeds mixed with D-Pen at various concentrations at (a) 0 min and (b) 20 min, and their corresponding (c) UV-Vis absorption and (d) CD spectra after 20 min; Figure S3: (a) UV-Vis absorption and (b) CD spectra of L-Se seeds mixed with L-GSH at various concentrations after 20 min; Figure S4: CD spectra of (a) the mixture containing 9 kinds of interferents (including Zn^2+^ and Cu^2+^) and L-Cys; and (b) the mixture containing 7 kinds of interferents (without Zn^2+^ and Cu^2+^) and L-Cys; Figure S5: CD spectra of L-Se seeds mixed with L-Cys solution (20 μmol/L, black line) and D-Cys (20 μmol/L, red line) after 20 min; Figure S6: CD spectra of L-Se seeds (black line) and D-Se seeds (red line) mixed with L-Cys solution (20 μmol/L) after 20 min; Figure S7: Raman spectra of (a) L-Se seeds and (b) L-Se seeds mixed with D-Pen (20 μmol/L) after 20 min.
