# Supplementary material for: Two Signals from One Event: Exploiting Intrinsic Dual-Functional Selenium Nanomaterials for Signal-On Chiroptical and Colorimetric Sensing of Free Thiols
Source: Biosensors (Basel). 2026 Sep 3;16(9):493. doi: 10.3390/bios16090493 (PMC13604616; doi:10.3390/bios16090493)
Supplement: Supplementary file 1 [file biosensors-16-00493-s001.zip › biosensors-4516572-supplementary.pdf]

biosensors

Article

Two Signals from One Event: Exploiting Intrinsic Dual-Functional Selenium Nanomaterials for Signal-on Chiroptical and Colorimetric Sensing of Free Thiols

Jiayang Gao [1] and Xinling Liu [2]*

1. College of Food Science and Technology, Shanghai Ocean University, Shanghai 201306, China; 2334202@st.shou.edu.cn
2. The Education Ministry Key Lab of Resource Chemistry, Shanghai Frontiers Science Center of Biomimetic Catalysis, College of Chemistry and Materials Science, Shanghai Normal University, Shanghai 200234, China
* Correspondence: xlliu@shnu.edu.cn

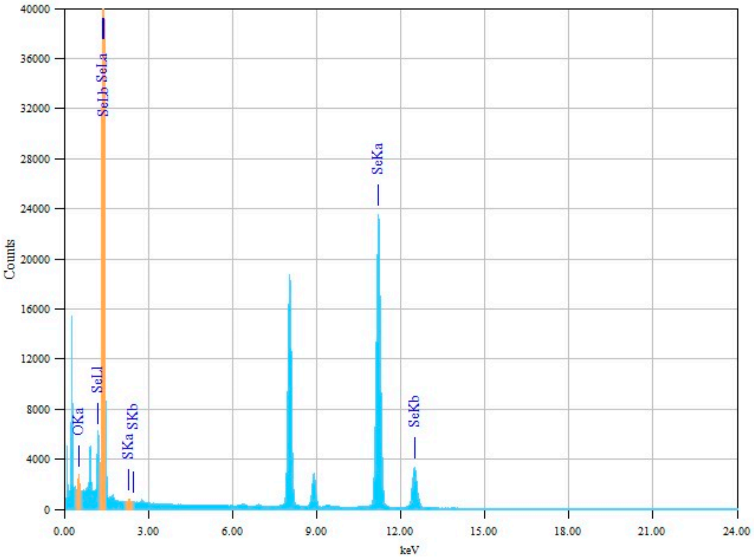

Figure S1. EDS spectrum of L-Se seeds (the atomic ratio of Se, S, O is 97.49%, 0.27%, and 0.38%, respectively).

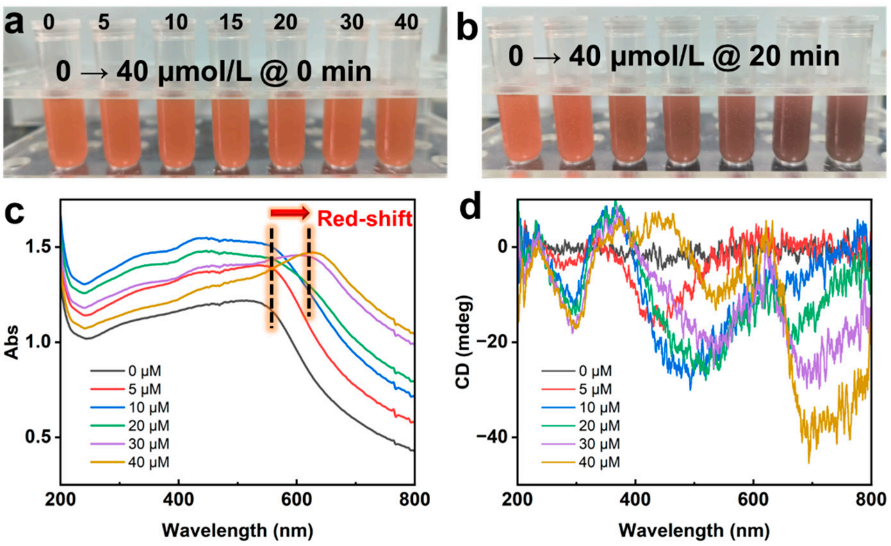

Figure S2. Photographs of L-Se seeds mixed with D-Pen at various concentrations at (a) 0 min and (b) 20 min, and their corresponding (c) UV-Vis absorption and (d) CD spectra after 20 min.

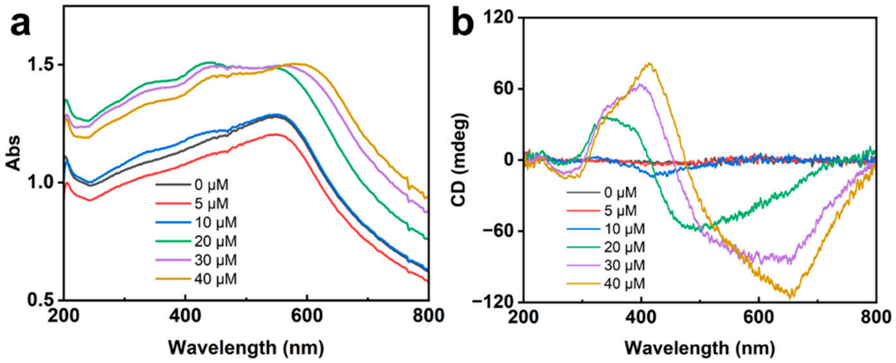

Figure S3. (a) UV-Vis absorption and (b) CD spectra of L-Se seeds mixed with L-GSH at various concentrations after 20 min.

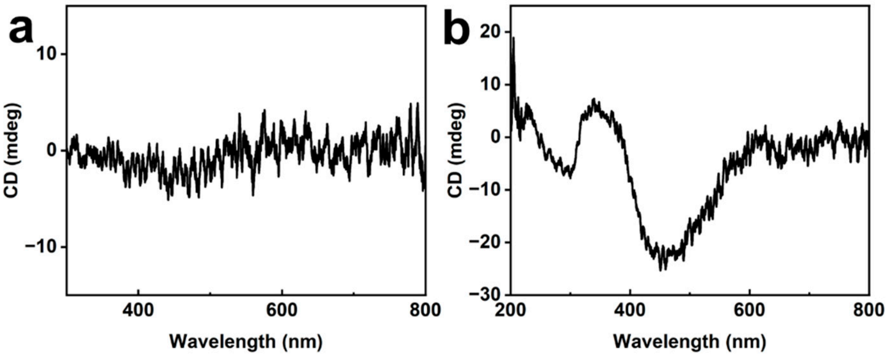

Figure S4. CD spectra of (a) the mixture containing 9 kinds of interferents (including Zn^{2+} and Cu^{2+}) and L-Cys; and (b) the mixture containing 7 kinds of interferents (without Zn^{2+} and Cu^{2+}) and L-Cys.

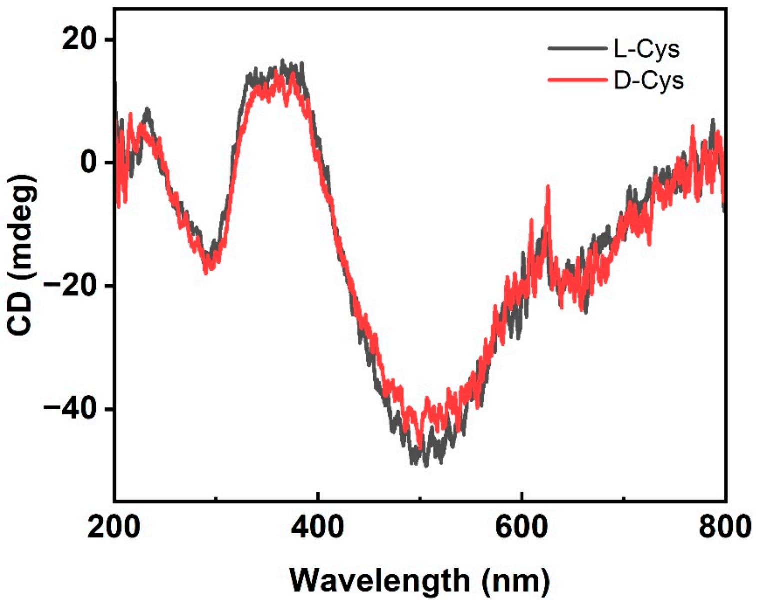

Figure S5. CD spectra of L-Se seeds mixed with L-Cys solution (20 μmol/L, black line) and D-Cys (20 μmol/L, red line) after 20 min.

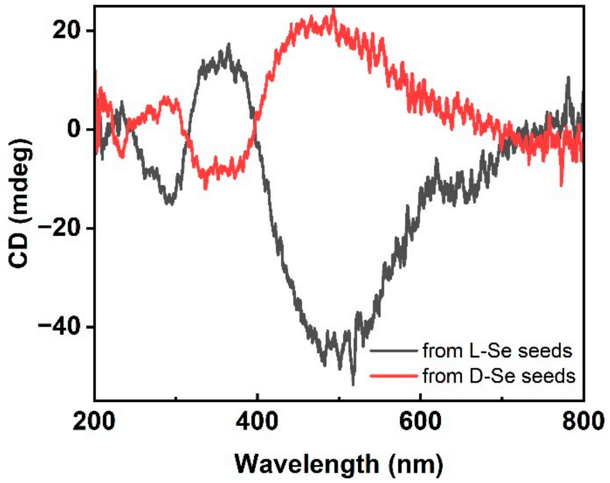

Figure S6. CD spectra of L-Se seeds (black line) and D-Se seeds (red line) mixed with L-Cys solution (20 μmol/L) after 20 min.

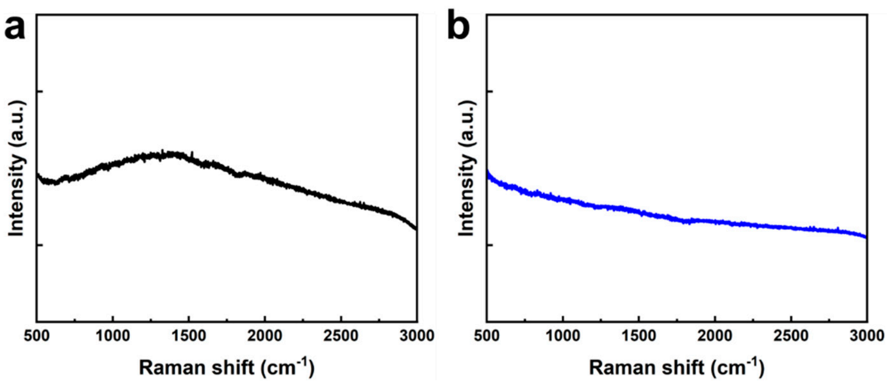

Figure S7. Raman spectra of (a) L-Se seeds and (b) L-Se seeds mixed with D-Pen (20 μmol/L) after 20 min.
